# Assessing the active living environment in three rural towns with a high proportion of Native Hawaiians and other Pacific Islanders

**DOI:** 10.15171/hpp.2017.25

**Published:** 2017-06-14

**Authors:** Siosaia F Hafoka

**Affiliations:** 1960 East-West Road, Honolulu, Hawaiʻi 96822, USA

**Keywords:** Active living, Built environment, Rural, Rural health

## Abstract

**Background:** Existing literature on the built environment and physical activity in rural areas is very limited. Studies have shown that residents in rural areas are less likely to meet physical activity requirements than their counterparts living in urban and suburban areas. They are also less likely to have access to amenities and programs that promote physical activity. This study seeks to fill gaps in the literature by assessing the built environment in three rural towns in Hawaiʻi that have a high proportion of Native Hawaiians and other Pacific Islanders.

**Methods: ** The Rural Active Living Assessment (RALA) tools will be used to assess the built environment. The RALA has three components – Policy and Program Assessment (PPA), Town Wide Assessment (TWA), and Street Segment Assessment (SSA) which will be used to provide a comprehensive assessment of the active living environment. Assessments were completed in September and October 2016.

**Results:** One assessment was completed in each town for the TWA and PPA. The SSA was completed with 60 segments (20 from each town).

**Conclusion:** The RALA tools identified supports in these three rural towns. The assessment also identified barriers and gaps – especially with the town and school polices of each town.

## Introduction

The World Health Organization (WHO) defines physical activity as “any bodily movement produced by skeletal muscles that require energy expenditure.”^1^ Some activities that meet this definition are walking, bicycling, swimming and playing in sports. The four most common domains used to capture physical activity are occupational, transport, household and leisure.^2,3^

The Center for Disease Control and Prevention (CDC) recommends 150 minutes of moderate-intensity aerobic activity (i.e., brisk walking) every week and muscle-strengthening (activities that increases muscle strength, power, endurance or mass) activities on 2 or more days a week that work all major muscle groups (e.g., legs, chest, shoulders and arms).^4^ Native Hawaiian and other Pacific Islanders (NHPI) are among the most obese people in the world. They also suffer from the highest rates of diabetes in the world.^5,6^ They also suffer from the highest rates of diabetes in the world.^7^ These, and other non-communicable diseases (NCDs) account for more than 70 of deaths among NHPI.^7^

Physical activity has been shown to reduce the risk of NCDs such as cardiovascular disease, diabetes, obesity and hypertension.^8,9^ Very low rates of NHPI meet physical activity recommendations.^10-12^ Nearly half of all NHPI (48.1%) do not meet the physical activity recommendations.^13^ Physical activity programs have been implemented throughout the Pacific to try and combat physical inactivity and prevent NCDs.^14^ Even with the implementation of physical activity programs, progress towards improving health outcomes and increasing opportunities for physical activities among NHPI has been minimal.^5^

As society moved away from farming and agriculture, physical activity related activities reduced. Technological advancements and changes in modes of transportation reduced the need of physical activity and manual labor. Urbanization, changes in diet and physical activity behaviors have all contributed to the rise of NCDs.^15,16^

With an increase in NCDs and an increase in physical inactivity, there has been a recent movement to examine the relationship of the built environment and health.^17-19^ The built environment encompasses zoning, parks, buildings and transportation infrastructure.^20^ Specific domains and characteristics in the built environment have been associated with NCDs and physical activity behaviors.^21,22^

Most studies examining the built environment and physical activity behaviors have focused on urban areas.^17^ Existing literature indicate that characteristics of the built environment can increase physical activity in urban areas.^23^ A literature review in 2015 identified very few studies that targeted rural areas, and of those studies, even fewer have targeted minority or indigenous populations.^24-26^

The aim of this study was to assess the built environment, and existing policies and programs of three rural towns in Hawaiʻi that have a high proportion of NHPI. To the author’s knowledge, no previous studies have assessed the built environment among NHPI in rural areas. Completing this assessment will help to fill that gap in the literature.

The social ecological model suggests that multiple levels and environments can influence health behaviors.^27^ These levels include the individual, social, physical and policy environments. This social ecological model was adapted to assess how the different environments can influence physical activity.^28^

**Figure 1:**
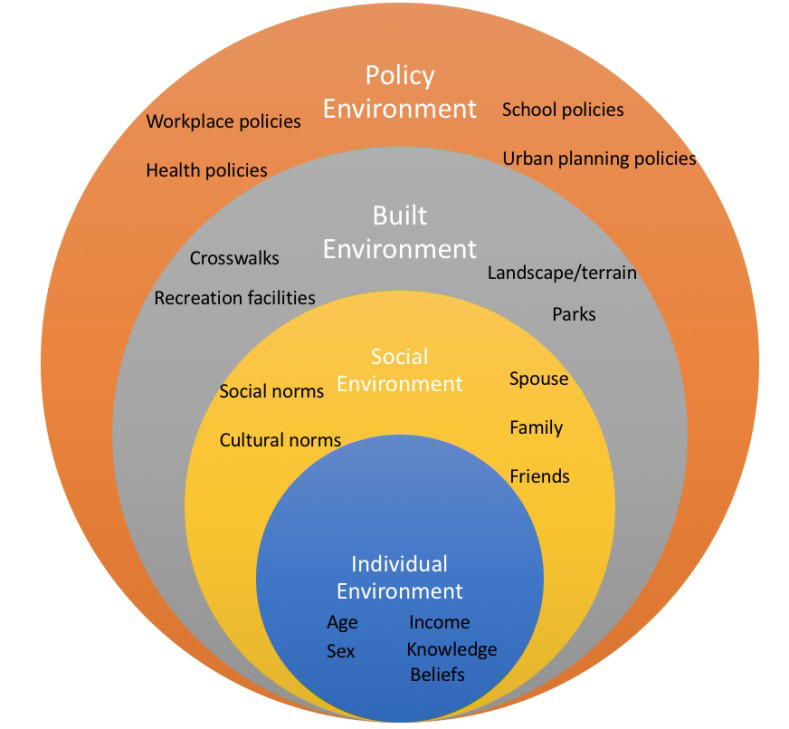
Social ecological model. Adapted from Sallis et al, 2006.

At the center of the theory is the individual (Figure 1). Factors that influence the individual at this level may include age, sex, income, knowledge and beliefs. Surrounding the individual is the social environment. The social environment has been shown to greatly influence an individual’s behavior.^29,30^ The social environment includes social and cultural norms, social support groups (church groups, teammates, coworkers). Surrounding the social environment is the physical or built environment. Recreational amenities such as parks, exercise equipment, bike paths and swimming pools all make up the built environment. The built environment also includes busy highways, dark streets and sidewalks or walkways. The policy environment encompasses all other environments in the adapted social ecological model. This study used the same framework to assess the current political, built and social environments of three rural towns in Hawaiʻi.

## Materials and Methods

Three rural towns in Hawaiʻi were selected for this assessment. The towns were assessed in September and October 2016. Street segments for the RALA were also assessed in the same months, on a non-holiday weekday between the hours of 8:00 am and 5:00 pm. The sample for this study was selected primarily because of its location, and the high proportion of NHPI living in the selected areas. A two-lane road connects the three towns with more urban areas of the island. The selected towns are described in Table 1. By population, Town 1 is the smallest of the three with 3292; Town 2 has the most residents with 6419. Town 3 has the highest proportion of residents who self-identify as NHPI 70.2% of total residents. Town 2 has the lowest proportion of NHPI residents, however, that proportion is still more than half of the current residents (51.1%). Income levels in Town 1 ($61 250) and Town 3 ($65 265) are lower than the state median ($68 201). All three towns have a higher percentage of people living under the poverty level than the state.

Rural areas have facilitators and barriers to physical activity that are different than those in urban areas.^31-34^ Access to physical activity facilities may be easier in some areas. Time may be a factor for some populations where they do not have to spend so much time traveling to and from work. These are some examples that reflect the importance of filling gaps in the literature to include assessments of the built environment in rural areas.

### Rural Active Living Assessment tools

The Rural Active Living Assessment (RALA) tools were developed because of a need to provide an assessment tool for rural areas.^35^ The RALA tools were made to assess different domains that are more likely to be found in rural areas.^36^ Similar studies assessing the rural active living environment have also used the RALA tools.^26,37^

The RALA consists of three assessment tools. The Street Segment Assessment (SSA) examines selected street segments within the town boundaries. The SSA has a total of 28 items that identify walkability (available sidewalk, buffers, and shoulder conditions), safety (e.g., street and pedestrian lighting), road features and land use. Examples of SSA items include: “Choose one option that best describes the sidewalks in the segment” (responses: Sidewalks are found on both sides of the street, one side of the street, intermittent, footpath only, or none); “Safety features” (responses: traffic light, stop signs, yellow school flashing lights, speed bumps, public lighting or none).

### Rural Active Living Assessment scoring

The Town Wide Assessment (TWA) tool uses 33 items to identify town demographics, school location, biking/hiking trails, public parks and playgrounds, water activities and other recreational facilities. TWA items include “There is a hiking or walking trail” (responses are “Yes, within 3 miles of town center,” [8 points] “Yes, 5-15 miles of town center,” [5 points] and “No” [0 points]); “Public use swimming pool” (responses are “Yes, within 15 miles of town center,” [4 points] or “No” [0 points]).

The Policy and Program Assessment (PPA) tool has 20 items to identify existing town programs and policies, and school programs and policies that promote physical activity. Examples of PPA items are: “Town has a public recreation department” (responses are “Yes” [10 points] or “No” [0 points]); There are “Walk to School” programs or other programs that encourage children to walk or bike to school” (responses are “Yes” [15 points] or “No” [0 points]).

The RALA tools also have a codebook and scoring tool to assist in the active living assessment. Domains from each area in the TWA are scored individually and can then combined for a total with the lowest possible a 0, and the highest possible score is 100 points. The PPA is also scored individually by school program and policies, and town program and policies. Those scores are also combined to provide a score totaling between 0 and 100. There is no existing scoring guide for SSA, and Table 2 reflect the number of existing facilities or amenities within each selected segment. The SSA simply provides a frequency of amenities and facilities of each street segment.

Data for the SSA were collected by walking each segment at least twice and checking each item. Street segments were completed between normal business hours of 8 am and 5 pm on a non-holiday weekday. Items for the PPA were answered by the researcher and then confirmed with a school and recreational official. Items from the TWA can be completed with publicly available data (e.g. maps) and was confirmed with local community members. Analysis of the data was done using SPSS (version 23, IBM Inc., Armonk, New York, USA). Raw scores were compared by town and domain. For further analysis, Fisher’s exact tests were performed to identify any significant frequency or score differences of amenities and facilities between towns. Significance levels were set at an alpha of 0.05.

## Results

The TWA scores individual domains in the town, and provides an overall town-wide score for available physical activity amenities (Table 3). Total scores for TWA ranged from 59 to 77. Two towns have a public park operated by the city and county government. Each of the three towns had access to at least one public beach. All three towns scored the highest possible in the Trails domain (20 points possible).

The PPA, as with the TWA, provides domain-specific scores and an overall score for the town’s physical activity programs and policies (Table 4). The highest possible score was 100 points. The scores ranged from 28 to 48 with Town 1 scoring the lowest and Town 3 scoring the highest of the three towns. There were no town policies and each town received a score of zero in that domain. Town 2 received the highest score in the School Programs domain.

A total of 60 SSA were completed; 20 from each town in the study. Unlike the TWA and PPA, the SSA portion of the RALA tools is not scored. The items in the SSA in each town were individually examined (Table 2). Fisher’s exact tests were performed for segment features and amenities by town. There was a significant difference between the frequency of sidewalk shoulders and buffers between the three towns (*P* < 0.00).

## Discussion

The lack of town policies to promote physical activity were obvious in the PPA scores. All three towns scored a zero when assessing the Town Policy domain. These scores are similar to a previous study that has used the RALA to assess towns in the southern US.^37^ The same study also reported that several towns did not have any existing town polices and also scored very low or zero. It is also important to consider that the PPA only has one item that was used to identify the Town Policy: Town has policy requiring bikeways/pedestrian walkways in new public infrastructure projects (response: Yes [10 points] or No [0 points]). Adding more items to the PPA or using another policy assessment tool may change the policy environment score of the three towns.

Town 2 scored the highest in the School Programs domain. All three towns offer programs that promote physical activity. Organizers of local youth sports leagues include the American Youth Soccer Organization (AYSO), Police Activities League (PAL), and Pop Warner, and recreational programs offered by the Honolulu City & County. The fees for these programs ranges from $0 (free) to $230. Some fees may not be affordable for everyone in these communities. Organizations may consider using a sliding scale for individuals/families so that these programs can be accessible to the entire community. Studies examining accessibility to physical activity amenities have also recommended using a sliding-scale fees.^38,39^

Of the 60 segments audited in the SSA, only three did not have any safety features (traffic light, stop sign, school flashing light, speed bump, or public lighting). Town 2 had the highest proportion of crosswalks (65%), sidewalks on both sides of the street (30%), sidewalk buffers and shoulders (45%), and route connections (40%). Town 2 also has marked bike lanes in selected street segments, and Towns 1 and 3 did not have any bike lanes. With sidewalks and crosswalks and a bike lane being more available in Town 2, the elementary school may be more likely to promote programs that encourage walking and biking to school.

A strength of this study is that this used an assessment tool made specifically for rural towns with a population of less than 10 000 residents. The RALA tools take a comprehensive approach and audits the town and school policies, as well as features and available amenities with the town.

It should also be noted that other studies have used the RALA tools.^26,27^ However, this is the first study to use the RALA in towns that have a high proportion of NHPI. There were limitations to this study. Since only three towns were assessed, results and findings may not be generalizable to all rural towns in Hawaii. Another limitation to this study is that there was one auditor for the SSA portion. Future research should include an assessment of community physical activity behavior and perceptions to identify possible associations with the results from this study.

The 2009 the State of Hawaii adopted Complete Streets, and in 2012 the Honolulu City & County adopted a Complete Streets policy and ordinance. Although the City & County “is taking aggressive steps to implement Complete streets,” none of the 16 study sites on Oahu are in small rural towns.^40^ Findings from the 16 urban areas may not be generalizable to rural areas, as there are significant differences – infrastructure, transportation, zoning, health status and risks.^41^

## Conclusion

The RALA tools can provide a comprehensive assessment of the built environment and policy environment of rural towns. Similar to previous studies, this study has identified both supports and barriers in the built and political environments (town and school policies) of a rural community. The results can be used as a “baseline” in the future for policy makers and community members to make improvements or modifications to the existing built and policy environments.

In order to create a healthy policy environment, and built environment for physical activity, collaborative efforts must be done across different sectors and organizations – policy makers, school officials, local leaders, and community members together. One example of how collaborative efforts have already happened, is that there have been bike paths and bike lanes constructed on private property and private roads. These amenities allow for community members to use bicycles in marked lanes, and bike paths for getting to work, school or to exercise. More work with active living should focus on areas with high proportions of NHPI and other minority groups, to reduce and eliminate health disparities.

## Ethical Approval

This study was approved by the University of Hawaiʻi at Mānoa’s Institutional Review Board prior to its initiation.

## Competing interests

No funding conflicts or other conflicts of interest to declare.

## Author’s contribution

SFH proposed the study, collected the data and prepared the manuscript for publication.

**Table 1:** US Census characteristics of rural Hawaii towns included in study

	**Town 1 No. (%)**	**Town 2 No. (%)**	**Town 3 No. (%)**	**Native Hawaiian (%)**
Population	3292	6419	5555	1 360 301
Native Hawaiian and Other Pacific Islander	59.5 (1,960)	56.8 (3,292)	70.2 (3904)	25.7 (350 288)
High school graduate or higher	2285 (87.4)	6009 (97.9)	3625 (87.4)	1 233 793 (90.7)
Median household income (dollars)	61 250	86 731	65 625	68 201
Persons below poverty level (%)	491 (14.9)	847 (13.2)	717 (12.9)	152 353 (11.2)

Source:  https://factfinder.census.gov/.

**Table 2:** Street Segment Assessment characteristics

**Variables**	**Town 1 (n = 20)**	**Town 2 (n = 20)**	**Town 3 (n = 20)**	***P*** ** value**
Commercial features	4 (20%)	2 (10%)	2 (10%)	0.56
Public/civic features	11 (55%)	5 (25%)	8 (40%)	0.17
Public playground	3 (15%)	2 (10%)	1 (5%)	0.57
Sidewalks (both sides of street)	0 (0%)	6 (30%)	2 (10%)	0.06
Sidewalk (one side of street)	3 (15%)	3 (15%)	1 (5%)	0.27
Sidewalk shoulder/buffers	1 (5%)	9 (45%)	2 (10%)	0.00^a^
Safety features (street lights)	18 (90%)	19 (95%)	20 (100%)	0.76
Crossing signals	1 (5%)	1 (5%)	0 (0%)	0.59
Crosswalks	7 (35%)	13 (65%)	2 (10%)	0.00^a^
Connectivity	2 (10%)	8 (40%)	3 (15%)	0.04^a^

No. (%) is reported. ^a^ *P* < 0.05.

**Table 3:** Town Wide Assessment scores

**Domain (max score)**	**Town 1**	**Town 2**	**Town 3**	**Overall mean (SD)**
School location (15)	15	6	6	9 (5.1)
Trails (20)	20	20	20	20 (0.0)
Parks and playgrounds (25)	23	18	26	22.3 (4.0)
Water activities (10)	5	5	5	5 (0.0)
Recreation facilities (30)	14	12	7	11 (3.6)
Total (100)	77	59	64	66.6 (9.2)

**Table 4:** Policy and Program Assessment score

**Domain (max score)**	**Town 1**	**Town 2**	**Town 3**	**Overall Mean(SD)**
Town policies (10)	0	0	0	0
Town programs (30)	18	8	18	14.6 (5.7)
School policies (30)	0	15	15	10 (8.6)
School programs (30)	10	25	10	15 (8.6)
Total	28	48	43	39(10.0)
